# Stiffening of the nucleus pulposus upon axial loading of the intervertebral disc: An experimental in situ study

**DOI:** 10.1002/jsp2.1005

**Published:** 2018-03-15

**Authors:** Steven V. Beekmans, Kaj S. Emanuel, Theodoor H. Smit, Davide Iannuzzi

**Affiliations:** ^1^ Department of Physics and Astronomy Vrije Universiteit Amsterdam Amsterdam Netherlands; ^2^ LaserLab Amsterdam Vrije Universiteit Amsterdam Amsterdam Netherlands; ^3^ Department of Orthopaedic Surgery VU University Medical Center (VUmc) Amsterdam Netherlands; ^4^ Amsterdam Movement Sciences VU University Medical Center (VUmc) Amsterdam Netherlands; ^5^ Department of Medical Biology Academic Medical Center (AMC) Amsterdam Netherlands; ^6^ Department of Orthopedic Surgery Academic Medical Center (AMC) Amsterdam Netherlands

**Keywords:** in situ viscoelastic properties, intervertebral disc, mechanical loading, minimally invasive microindentation, nucleus pulposus

## Abstract

Mechanical loading is inherently related to the function and degeneration of the intervertebral disc. We present a series of experiments aimed at measuring the effect of a loading/unloading cycle of the intervertebral disc on the mechanical properties of the nucleus pulposus. The study relies on our new minimally invasive microindenter, which allows us to quantify the storage and loss moduli of the nucleus pulposus by inserting an optomechanical probe in an intact (resected) intervertebral disk through the annulus fibrosis via a small needle. Our results indicate that, under the influence of compressive loading, the nucleus pulposus exhibits a more solid‐like behavior.

## 1 INTRODUCTION

The nucleus pulposus (NP), the water‐rich gelatinous center of the intervertebral disc (IVD), primarily bears the pressure on the spine and plays a major role in the degeneration of the IVD. The NP is strongly confined between the annulus fibrosis (AF) and the 2 vertebral end‐plates (VEP) of the IVD.^1^ The NP contains large concentrations of negatively charged proteoglycans (PGs), which cause it to retain water and maintain its swelling pressure.^2^ Surrounding the PGs, sparsely arranged collagen fibrils serve as a supporting matrix. Due to the limited amount of structural components inside the NP, its mechanical properties are dependent on the amount of PGs and water in the NP.^3^ As the amount of water in the IVD slowly changes with loading,^4^ the mechanical properties likely depend on the loading history.

During a 24‐hour day and night period, the spine is exposed to a cycle of mechanical axial loading and unloading. Mechanical loading can have various direct and indirect effects on the IVD, including, in severe cases, disc degeneration.^5^ Although the metabolic effects of the NP of disc loading have been described extensively,^6^ little research effort has been spent investigating the effect of loading on the mechanical properties of the NP. This may be ascribed to the complex confinement of the NP within the IVD. Conventional mechanical characterization instruments, such as rheometers, require extraction of the NP from the disc, which may result in damage caused by (1) the surgical procedure, (2) exposure to air or water, resulting drying or swelling, and (3) bulging due to the loss of confinement, all of which are known to alter the mechanical properties of the NP. The mechanical properties of the unconfined NP are thus dependent on the measurement protocol, as indicated by a large spread in elastic moduli in the literature values.^7^, ^8^, ^9^, ^10^, ^11^

Recently, our group presented a novel minimally invasive device that allows the user to measure the mechanical properties of biological tissue, such as its stiffness, at the tip of a rigid needle by means of microindentation.^12^ This method has been further improved to incorporate a full rheological analysis using an 18G needle—an approach dubbed as minimally invasive microindentation (MIMI).^13^ The small footprint of this device allows for minimally invasive measurements of the localized mechanical properties of a material beneath its surface. The ability to perform subsurface stiffness measurements is in sharp contrast with conventional mechanical testing instruments, the range of which is limited to the surface of a sample. Hence, this approach enables us to record the mechanical properties of the NP, in terms of the elastic and viscous modulus, while maintaining NP confinement, as presented in Reference^13^. Using our minimally invasive indenter, here, we present a full, in situ characterization of the mechanical properties of the confined NP, thus bearing the native confinement by the AF and VEP, during a cycle of mechanical loading and unloading of the IVD.

## 2 MATERIALS AND METHOD

### 2.1 IVD preparation

Isolated intact spines of skeletally mature female milk goats (age 3‐4 years) were obtained from a local butcher and processed immediately after isolation. To prepare specimens for testing, individual discs were separated from the spine with an oscillating saw, maintaining 2 to 5 mm of flat endplate on both sides. Subsequently, discs were brushed clean, rinsed and stored in physiological saline‐soaked gauzes at −20°C. Before testing, discs were thawed in lukewarm saline water for 30 minutes. The typical cross‐sectional area of the discs was 900 mm^2^.

### 2.2 Minimally invasive microindentation

**Figure 1:**
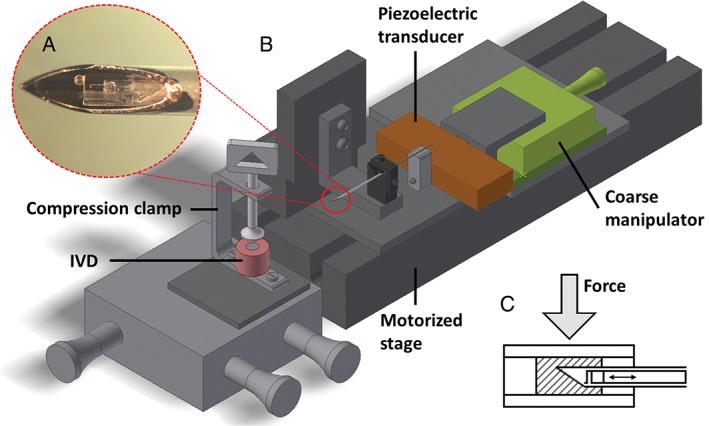
Schematic view of the experimental setup. See Reference 13 for details. A, Close‐up image of the minimally invasive microindentation (MIMI) probe at the tip of an 18G needle. B, Schematic of the probe, mounted on a long‐range piezoelectric translator (orange) which is attached to a manual translation stage (green). The probe is housed in a thin needle, fixed on a motorized linear stage. The intervertebral disc (IVD) (pink) is clamped in front of the needle between a pivoting and a flat plate and can be positioned in 3 dimensions. C, Schematic of the needle inside the IVD. Load can be applied on the IVD by increasing the compression force. The nucleus pulposus (NP) is highlighted by diagonal lines. Not to scale

MIMI is based on an optical force transducer at the tip of a thin needle and employs the bending of a micromachined cantilever to infer the viscoelastic properties of a sample by means of dynamic mechanical analysis (DMA).^12^, ^14^, ^15^ The details of the device are described in previous work.^13^ The cantilever, the bending of which is monitored via a Fabry‐Pérot cavity using a cleaved single mode optical fiber, is equipped with a spherical tip with a diameter of around 200 μm. The probe is mounted in the lumen of an 18G needle and can be retracted to a safe distance from the needle tip (Figure 1A). By means of a long‐range piezoelectric transducer (P‐602.5L8; Physike Instrumente GmbH, Karlsruhe, Germany), mounted at the proximal part of the needle, fine positioning of the probe can be achieved. The needle is fixed on a motorized linear stage (LTS300; Thorlabs GmbH, Newton, New Jersey), which is used for needle insertion (Figure 1B).

Before installation in the needle, the spring constant of newly fabricated probes is calibrated using a customized calibration method for cantilevers with interferometric readouts.^16^ Effective cantilever spring constants varied between 60 and 70 N/m.

To obtain a mechanical characterization of the NP with the microindenter, we have made use of dynamic indentation.^15^ DMA is based on load‐ or indentation‐controlled oscillations applied on top of a fixed static load. Precise control of load (ie, cantilever bending) or indentation depth is crucial to ensure consistent stress or strain, respectively, in the sample with each indentation. We have performed our dynamic indentations in load‐control mode. After contact with the NP is confirmed, the probe is moved forward (thus bending the cantilever) until the static load applied on the tissue matches the preset value (∼300 μN). To allow the tissue to equilibrate, we maintain the fixed static load for 60 seconds. Afterwards, the load is swept over 5 frequencies (oscillations with 5 periods each), logarithmically spaced and in increasing order between 0.5 and 10 Hz. The amplitude of the oscillations is set to 10 μN in order to (1) keep the indentation depth in the viscoelastic regime and (2) ensure that indentation depth is much smaller than the bead radius (max indentation depth ∼40 μm). We obtain shear storage and loss moduli (*G*′ and *G*″) from the dynamic indentation results by applying an analytical solution for DMA.^14^

### 2.3 Measurement protocol

Prior to each experiment, an indentation procedure on glass is performed to account for a possible geometrical offset between the position of the cleaved optical fiber and that of the spherical tip. IVDs are clamped on top of a 3‐axis micromanipulation stage (MAX312D; Thorlabs GmbH, Newton, New Jersey) and are positioned in front of the needle (Figure 1). The clamping force is minimized to prevent initial axial stress on the disc. Before the first insertion, unloaded discs are probed with a 21G hypodermic needle (Neolus NN‐2138R; Terumo, Tokyo, Japan) to locate a suitable insertion trajectory. Subsequently, the 18G needle (manufactured in‐house from a stainless‐steel capillary with diameter: 1.3 mm, wall thickness: 0.1 mm, Salomon's metalen b.v.), housing the microindenter, is inserted 1 to 3 mm inside the NP through the AF (Figure 1C). After reaching the target location, insertion of the needle is stopped and the probe is carefully advanced using a coarse long‐range transducer until contact with the tissue is observed. Upon contact with the NP tissue, the probe is retracted for 100 μm and dynamic indentation is started. In case of contamination of the lumen of the needle, indicated by premature contact with the tissue, the probe and the needle are retracted and the lumen is cleaned. Five frequency scans are obtained for each insertion.

To study the effect of IVD loading on the mechanical properties of the NP, the whole IVD is compressed rapidly (∼2 seconds) in the clamp until a strain on its total height of 10% is reached, corresponding to approximately 1000 N. The top plate of the clamp allows for sufficient pivot to ensure axial loading. After 30 minutes, the compressed disc is reinserted with the needle (ie, in the same location) and NP mechanical properties are recorded according to the same protocol. Afterwards, the disc is unloaded and, after 30 minutes, the same location in the NP is tested once more. Cycles of 30 minutes have been chosen to allow for observable changes in the sample while avoiding significant drift in the measurement system. The compressive load is not continuously monitored and, therefore, a small amount of stress relaxation could not be prevented. This is neglected in the further analysis, as the aim of this study is to observe the effect of a significant load on the IVD to the dynamic elastic moduli of the NP. The data for *G*′ and *G*″ were assumed to be distributed normally. Statistical differences between loading states were investigated by means of a paired Student's *t* test.

## 3 RESULTS

In this study, 6 sites (2 per disc), all in the center of the NP, were successfully characterized in terms of storage and loss moduli during a cycle of loading and unloading. Each site was approached from a different angle but it was ensured that the penetration depth in the IVD was always the same. Needle insertions were performed before, during, and after loading on the IVD. Five dynamic indentations were performed for each needle insertion. The time between states of loading/unloading was set to 30 minutes for each cycle.

**Figure 2:**
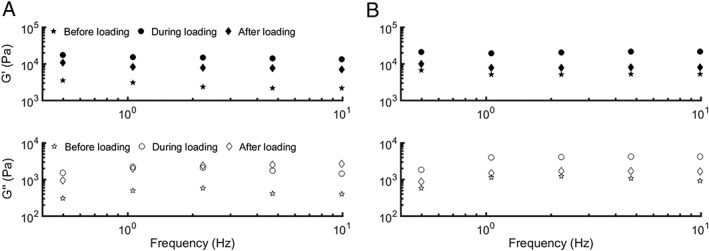
Quantitative comparison of the viscoelastic behavior of the nucleus pulposus (NP) before, during, and after loading of disc 1, as measured in situ for 2 central sites in the NP (different insertion angle, same penetration depth) in the same disc (A and B). Frequency‐dependent storage (closed symbols) and loss (open symbols) moduli are averaged over 5 frequency sweeps at each location. An increase in *G*′ and *G*″ during the loaded state can be observed for both sites. For site (A), *G*′ and *G*″ present a low degree of recovery, contrary to site (B), where a high degree of recovery can be observed

In Figure 2, we report the in situ frequency‐dependent storage and loss moduli of the NP measured before, during, and after loading of disc 1. We observed a systematic increase in both *G*′ and *G*″ in the NP after loading of the IVD. After releasing the load, the NP showed recovery in all the measured sites, albeit to a variable extent, as illustrated by the example in Figure 2. Figure 2A shows a low degree of recovery, whereas in Figure 2B an example of a high degree of recovery can be seen. We report a minor upwards trend for both *G*′ and *G*″ with increasing frequency. This weak gel‐like rheological behavior has been reported earlier for porcine NP and can be related to the NP's physiological role as a shock absorber.^8^

**Figure 3:**
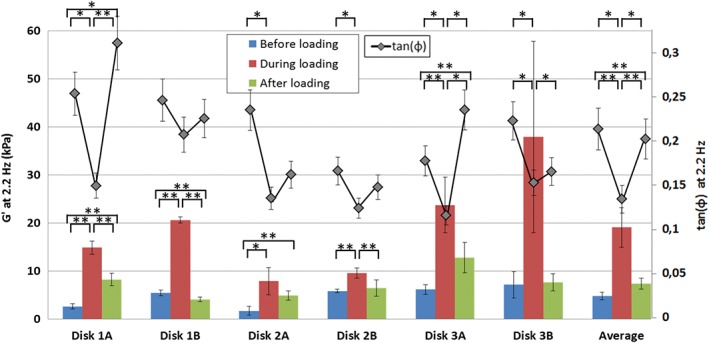
Storage modulus (*G*′) and tan(δ) at 2.2 Hz for 6 cycles of loading and unloading on 6 central sites in the nucleus pulposus (NP). Each bar or point represents an average of 5 measurements at 2.2 Hz, corresponding to those depicted in Figure 2 for disc 1A and 1B. Statistical differences in *G*′ between the different loading state are indicated directly above the bars (* indicates α < 0.05, ** indicates α < 0.01). Statistics for tan(δ) are shown at the top of the chart. All error bars are SDs

To illustrate the effect of IVD loading on the mechanical properties of the NP, in Figure 3 we show the average storage modulus at 2.2 Hz for each tested site in the NP before, during, and after loading, as well as the total average for *G*′ at 2.2 Hz. The storage and loss moduli of the unloaded NP varied slightly between the measured sites, as was expected with varying locations and discs. The total average *G*′ at 2.2 Hz of the unloaded NP was 4800 ± 800 Pa, in agreement with previous work—a comparison of *G*′ and *G*″ of the unloaded NP with literature values can be found in Reference 13. A systematic increase of *G*′ during loading can be clearly observed in Figure 3 for each of the sites, and is confirmed by the total average (19 100 ± 4100 Pa). An increase of *G*′, corresponding to a stiffening of the material, can be correlated to one of the main biological functions of the NP, namely maintaining the height of the IVD under compressive loading.^17^ An increase in whole disc compressive stiffness during axial loading has been reported repeatedly in the literature before.^18^, ^19^

## 4 DISCUSSION

The value of *G*′ of the NP, in most cases, did not recover to its unloaded level after releasing the load. Two possible explanations for the absence of full recovery are: (1) the recovery time of 30 minutes was not sufficient, or (2) the induced mechanical load was too high, causing irreversible damage to the NP. Mechanical overloading is associated with accelerated disc degeneration and it has been reported that disc cells, including the NP, respond to mechanical loading in a manner that depends on the magnitude, duration, and frequency of the loading.^20^ A recovery time of 30 minutes may be relatively short with respect to a physiological loading/unloading cycle of 24 hours.^19^ Performing measurements with a longer waiting time is, however, not ideal either. Using short cycles, the sample remains in similar conditions throughout the measurement. Moreover, by doing so, significant drift in the measurement system is avoided while, at the same time, a recovery time of 30 minutes enables observable differences in fluid content. For the same reason, we reduced the frequency sweep range to 0.5‐10 Hz and decided to limit the amount of frequencies in the sweep to 5, as measuring at longer timescales increases the influence of time‐dependent changes that occur in soft, hydrated tissues. The varying amount of recovery of the sites may also be attributed to the position of the disc in the spine,^8^ or to the location that was probed within the NP, as the strain is not uniform inside the NP during loading.^21^

In addition, in Figure 3, we report the value of tan(φ) (= *G*″/*G*′) during the loading cycle. Tan(φ), sometimes called the loss factor, quantifies the balance between the loss and storage moduli and can be seen as a weighted indicator of the viscosity of a material. A value for tan(ϕ) above unity indicates a more liquid‐like behavior, whereas a tan(ϕ) closer to zero indicates more solid‐like properties. The average tan(ϕ) value before and after compression of the disc was 0.21 ± 0.03 and 0.20 ± 0.03, respectively, indicating gel‐like behavior of the NP. During compression, the average tan(φ) dropped to 0.14 ± 0.03, indicating that the NP has lost a significant amount of liquid to the AF or VEP—no fluid outflow was observed during the loading of the disc—and thus demonstrates stiffening under compression. This is in line with the suggestions that the NP is a material with biphasic/poroelastic properties,^22^, ^23^ as the compression will induce a loss of water, increasing the solid to fluid ratio, and therefore shifting towards a solid‐like behavior. Furthermore, after the fluid is expelled, the solid matrix will stiffen to withstand the compressive forces.^24^ This observation is in sharp contrast with the traditional description of the NP as an incompressible rubber often used in computational modeling.^25^, ^26^

In this study, *G*′ and *G*″ are calculated using an indentation model, which is set in a viscoelastic framework. This analysis provides information on the mechanical behavior of the NP under compression in situ. However, the lack of a direct measurement of poroelastic properties is a limitation of this study. Poroelasticity is often described by means of finite element models which require a priori information.^27^, ^28^ In light of these methods, in Figures S1‐S3 , we present the complex modulus (*G* ^*^) and phase angle corresponding to Figures 2 and 3. By applying fitting methods one can obtain indirect information about poroelastic/biphasic properties.^27^, ^29^, ^30^ Following Reference 28, one can find direct evidence of poroelasticity by observing a shift in the characteristic frequency of tan(ϕ) with varying indentation depth. This frequency shift can be best observed when a continuous frequency sweep is applied to the sample. Due to the discrete amount of frequencies probed during DMA, the current study is limited to an analysis in a viscoelastic framework. Therefore, to assess the poroelastic behavior of the NP in more detail, future experiments could include denser frequency sweeps or employ a continuous sweep model, such as in Reference 28.

**Figure 4:**
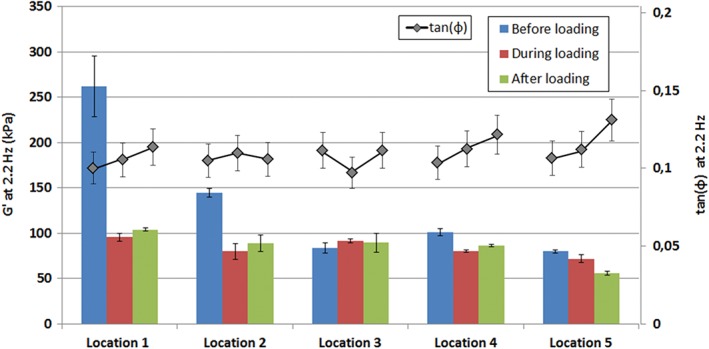
Reference experiment on a polymeric sample to test the influence of stress on the needle shaft on the dynamic analysis. Measurements of storage and loss moduli were performed according to the same protocol as the intervertebral disc (IVD) measurements. Each bar represents the average *G*′ of 5 measurements at 2.2 Hz. No significant deviation of *G*′ or tan(δ) was found during loading of the disc. All error bars are SDs

Loading of the IVD by means of compression results in a substantial amount of stress in the tissue, in particular in the AF. Once the needle has penetrated the AF, part of this stress may be transferred to the needle shaft. In order to assess whether the stress on the needle has an influence on the dynamic analysis we have designed a reference experiment, in which the needle is subjected to stresses comparable to those during a loading experiment, while the reference sample is insensitive to mechanical loading (we used a polymeric sample, prepared as in Reference 15). As the mechanical properties of the reference sample are insensitive to stress, the same results for *G*′ and *G*″ were expected before, during, and after loading. Figure 4 reports the average for *G*′ at 2.2 Hz of 5 frequency sweeps for 5 cycles of loading and unloading. From the absence of an increase in *G*′ during loading it can be concluded that stress on the needle shaft due to loading of the disc does not influence the dynamic mechanical analysis. Therefore, the findings of this study are not influenced by a systematic error induced by the measurement device.

In conclusion, in this study, we have recorded the in situ mechanical properties of the NP during a cycle of loading and unloading using our in‐house developed minimally invasive microindentation technique. A complete, in situ, mechanical characterization of the NP before, during, and after mechanical loading of the IVD showed an increase of the storage and loss moduli, and, more importantly, a decrease of tan(φ) after 30 minutes of axial loading. Recovery of the moduli was observed in all cycles 30 minutes after releasing the load, although only 50% of the sites recovered to the same level as before testing. Our results indicate stiffing of the NP during axial IVD compression, which is in line with the suggestion that the NP can also be described as a poroelastic material.

## Supporting information


**Figure S1** . Quantitative comparison of the viscoelastic behavior of the nucleus pulposus (NP) before, during, and after loading of disc 1, as measured in situ for 2 central sites in the NP (different insertion angle, same penetration depth) in the same disc (A and B). Complex (G*, closed symbols) and phase angle (open symbols) moduli are averaged over 5 frequency sweeps at each location. An increase in G* and a decrease in the phase angle during the loaded state can be observed for both sites. For site (A), G* presents a low degree of recovery, contrary to site (B), where a high degree of recovery of G* can be observed. In the phase angle, recovery can be observed at both sites.
**Figure S2**. Complex modulus (G*) at 2.2 Hz for 6 cycles of loading and unloading on 6 central sites in the nucleus pulposus (NP). Each bar or point represents an average of 5 measurements at 2.2 Hz, corresponding to those depicted in Figure S1 for disc 1A and 1B. Statistical differences in G* between the different loading state are indicated directly above the bars (* indicates *α* < .05, ** indicates *α* < .01). All error bars are SDs.
**Figure S3**. Phase angle at 2.2 Hz for 6 cycles of loading and unloading on 6 central sites in the nucleus pulposus (NP). Each bar or point represents an average of 5 measurements at 2.2 Hz, corresponding to those depicted in Figure S1 for disc 1A and 1B. Statistical differences in the phase angle between the different loading state are indicated directly above the bars (* indicates *α* < .05, ** indicates *α* < .01). All error bars are SDs.
