# Supplementary material for: Stiffening of the nucleus pulposus upon axial loading of the intervertebral disc: An experimental in situ study
Source: JOR Spine. 2018 Mar 15;1(1):e1005. doi: 10.1002/jsp2.1005 (PMC6686818; doi:10.1002/jsp2.1005)
Supplement: Supplementary file 1 — Figure S1 . Quantitative comparison of the viscoelastic behavior of the nucleus pulposus (NP) before, during, and after loading of disc 1, as measured in situ for 2 central sites in the NP (different insertion angle, same penetration depth) in the same disc (A and B). Complex (G*, closed symbols) and phase angle (open symbols) moduli are averaged over 5 frequency sweeps at each location. An increase in G* and a decrease in the phase angle during the loaded state can be observed for both sites. For site (A), G* presents a low degree of recovery, contrary to site (B), where a high degree of recovery of G* can be observed. In the phase angle, recovery can be observed at both sites. Figure S2. Complex modulus (G*) at 2.2 Hz for 6 cycles of loading and unloading on 6 central sites in the nucleus pulposus (NP). Each bar or point represents an average of 5 measurements at 2.2 Hz, corresponding to those depicted in Figure S1 for disc 1A and 1B. Statistical differences in G* between the different loading state are indicated directly above the bars (* indicates α < .05, ** indicates α < .01). All error bars are SDs. Figure S3. Phase angle at 2.2 Hz for 6 cycles of loading and unloading on 6 central sites in the nucleus pulposus (NP). Each bar or point represents an average of 5 measurements at 2.2 Hz, corresponding to those depicted in Figure S1 for disc 1A and 1B. Statistical differences in the phase angle between the different loading state are indicated directly above the bars (* indicates α < .05, ** indicates α < .01). All error bars are SDs. [file JSP2-1-e1005-s001.pdf]

Supplemental Information for Stiffening of the nucleus pulposus upon axial loading of the intervertebral disc: an experimental *in situ* study

Steven V. Beekmans[1,*], Kaj S. Emanuel[2], Theodoor H. Smit[3], and Davide Iannuzzi[1]

[1]Department of Physics and Astronomy and LaserLab Amsterdam, Vrije Universiteit Amsterdam, De Boelelaan 1085, 1081 HV, Amsterdam, Netherlands, s.v.beekmans@vu.nl

[2]Department of Orthopaedic Surgery, VU University Medical Center (VUmc), Amsterdam Movement Sciences, De Boelelaan 1117, 1081 HV, Amsterdam, Netherlands

[3]Department of Medical Biology and Department of Orthopedic Surgery, Academic Medical Center (AMC), Meiberdreef 9, 1105 AZ, Amsterdam, Netherlands

January 29, 2018

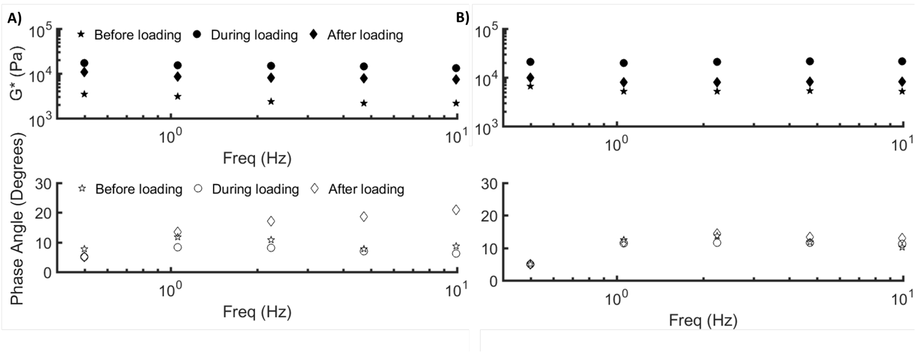

Figure S.1: Quantitative comparison of the viscoelastic behavior of the NP before, during and after loading of disc 1, as measured *in situ* for 2 central sites in the NP (different insertion angle, same penetration depth) in the same disc (A and B). Complex modulus (G^*, closed symbols) and phase angle (open symbols) moduli are averaged over 5 frequency sweeps at each location. An increase in G^* and a decrease in the phase angle during the loaded state can be observed for both sites. For site (A), G^* presents a low degree of recovery, contrary to site (B), where a high degree of recovery of G^* can be observed. In the phase angle recovery can be observed at both sites.

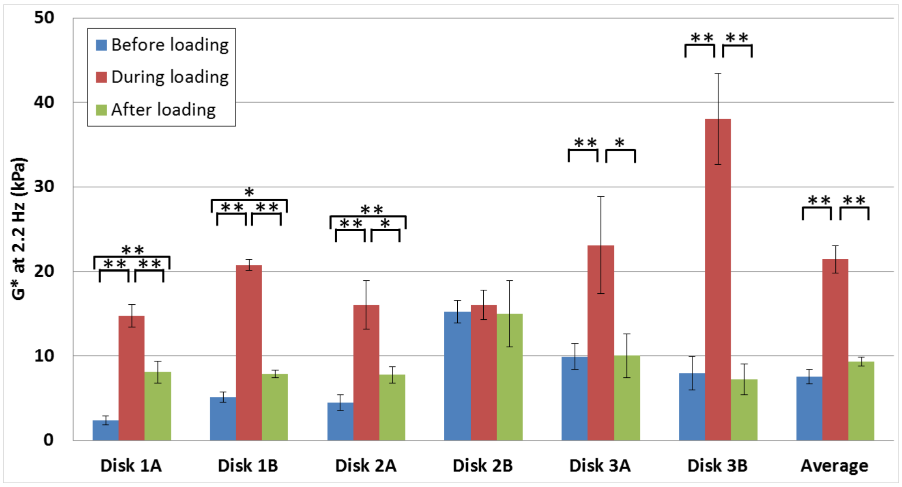

Figure S.2: Complex modulus (G^*) at 2.2 Hz for six cycles of loading and unloading on six central sites in the NP. Each bar or point represents an average of 5 measurements at 2.2 Hz, corresponding to those depicted in figure S.1 for disc 1A and 1B. Statistical differences in G^* between the different loading state are indicated directly above the bars (* indicates $\alpha < 0.05$, ** indicates $\alpha < 0.01$). All error bars are standard deviations.

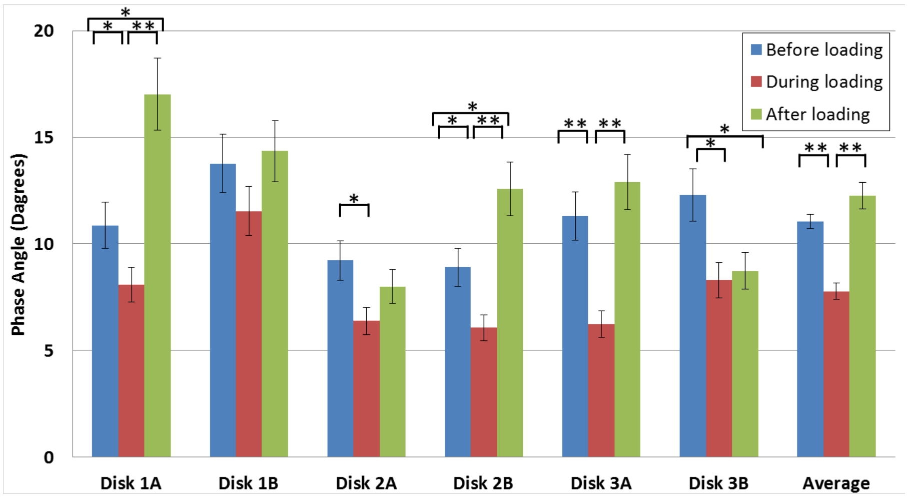

Figure S.3: Phase angle at 2.2 Hz for six cycles of loading and unloading on six central sites in the NP. Each bar or point represents an average of 5 measurements at 2.2 Hz, corresponding to those depicted in figure S.1 for disc 1A and 1B. Statistical differences in the phase angle between the different loading state are indicated directly above the bars (* indicates $\alpha < 0.05$, ** indicates $\alpha < 0.01$). All error bars are standard deviations.
